# Prevalence and Associated Factors of Urinary Incontinence Among Chilean Community-Dwelling Older Adults: A Cross-Sectional Study

**DOI:** 10.7759/cureus.77831

**Published:** 2025-01-22

**Authors:** Carolina Neira-Maldonado, Juan-Carlos Coronado, Mauricio A Álvarez, Rosario Negrón, Felipe Ponce-Fuentes, Iván Cuyul-Vásquez

**Affiliations:** 1 School of Kinesiology, Faculty of Medicine and Health Sciences, Major University, Temuco, CHL; 2 Department of Therapeutic Processes, Faculty of Health Sciences, Universidad Católica de Temuco, Temuco, CHL; 3 Dialysis Unit, Hospital Dr. Mauricio Heyermann Torres, Angol, CHL

**Keywords:** aged, cross-sectional study, prevalence, urinary incontinence, urination disorders

## Abstract

Background: Systematic reviews of the global prevalence of urinary incontinence (UI) have shown an underrepresentation of the South American population. The objective of this study was (i) to determine the prevalence and associated factors of UI among older Chilean community-dwelling older adults and (ii) to analyze the differences between older adults with and without UI.

Methods: This is an analytical cross-sectional study. A total of 144 community-dwelling older adults aged 60 years and above were eligible to participate in the study. Participants were asked to provide a range of data, including demographic data and anthropometric and self-reported measures. The prevalence of UI was estimated using the International Consultation on Incontinence Questionnaire-Short Form (ICIQ-SF). The analyzed factors included age, sex, weight, height, residence comorbidities, polypharmacy, cognitive function, and activity of daily living (ADL) functionality.

Results: One hundred eight older adults were included in the study. The median age of the participants was 69.0 ± 8.0 years, 84 (77.8%) were women, and 71 (65.7%) lived in an urban residence. The prevalence of UI in the total sample was 48.1% (n = 52). Stress, urge, and mixed types of UI were present in 75 (69.4%), 15 (14.3%), and 18 (16.3%) participants, respectively. Female sex, rheumatoid arthritis, and hypothyroidism were significantly more frequent in older adults with UI (P < 0.05). Correlations ranging from negligible to weak were observed between the presence of UI and the female sex (r = 0.25; P < 0.05), rheumatoid arthritis (r = 0.23; P < 0.05), and hypothyroidism (r = 0.35; P < 0.01).

Conclusions: In a sample of community-dwelling older adults, UI was present in 48.1% of the participants. The prevalence of UI was correlated with the female sex, rheumatoid arthritis, and hypothyroidism.

## Introduction

Urinary incontinence (UI) is defined as involuntary urine leakage [1]. Although not life-threatening, it is a problem with a negative impact on quality of life [1]. UI affects both sexes, although a higher prevalence has been reported in women (51.1% versus 13.9%) [2]. Historically, UI has affected 25% of the global population, with prevalence rates reaching up to 45% in women in certain countries and an estimated prevalence of 16% and 22% in older men and women, respectively [3]. Three types of UI have been defined: (i) stress, characterized by the involuntary urine loss during effort or physical exertion or, in general, during increased abdominal pressure (i.e., sneezing or coughing), (ii) urge, defined by concomitant urine loss or immediately after an urgency episode, and (iii) mixed, which is a combination of stress and urge UI [4]. This problem is more common in older adults, especially women, but is undervalued and scarcely studied [5].

There are many risk factors that affect UI in older adults, such as age, sex, and chronic health conditions [6-8]. As individuals age, normal age-related changes, including alterations in bladder function and pelvic floor support, can lead to an increased prevalence of UI [6]. Specifically, studies have shown that older adults have a higher incidence of UI, with rates significantly increasing to 24.3% among those over 75 years of age [9]. Another significant risk factor is sex, with women experiencing higher rates of UI [7]. Factors such as childbirth and hormonal changes associated with menopause contribute to this disparity between men and women in the prevalence and incidence of UI [7]. For instance, studies have indicated that multiparity and vaginal deliveries are strongly associated with the development of UI in women [5]. Obesity has also been identified as a critical risk factor, as excess weight can increase abdominal pressure and stress on the pelvic floor muscles, exacerbating UI symptoms [7]. Chronic health conditions also play a vital role in the risk of UI. Conditions such as diabetes mellitus, stroke, and neurological disorders have been associated with increased UI prevalence [8]. Diabetes mellitus, in particular, has been highlighted as a significant risk factor due to its effects on nerve function and bladder control [7]. Furthermore, functional disabilities, which are common in older adults, can limit mobility and the ability to reach the bathroom in time, further increasing the risk of UI [10]. Recognizing the biological, functional, and psychological factors linked to UI in older adults is crucial.

While UI is widely acknowledged as a disease affecting older adults, UI itself is viewed as a risk factor for falls and fractures in the older population, primarily because of its related metabolic disruptions and systemic inflammation [11]. Addressing UI risk factors through targeted interventions can significantly improve the quality of life for older adults with UI. However, systematic reviews of the global prevalence of UI have shown an underrepresentation of the South American population [12]. Furthermore, scarce and outdated data are available on the prevalence of UI and associated factors in Chilean older adults [13]. Consequently, the purpose of this study was to assess the prevalence and association between UI and a variety of demographic data, anthropometric measures, and self-reported questionnaires in community-dwelling older adults and to analyze the differences between older adults with and without UI. We expected that UI was common in older adults, and our hypothesis was that demographic data, anthropometric measures, and clinical factors may be associated with UI in this population.

## Materials and methods

Study design and setting

A cross-sectional analytical study was developed for Chilean community-dwelling older adults living in Vilcún City, Chile. A local health community center facility for older adults was used for the study evaluations. This center comprised a spacious area of 100 m² and two stations, each equipped with a chair, a table, a digital weight scale (BC-730, Tanita, Tokyo, Japan), and a wall stadiometer (201, Seca, Hamburg, Germany). The community center facility was available once a month for a period of eight hours (8:30 am to 16:30 pm) for the evaluation of participants. Two physical therapists performed the assessments, and each participant was scheduled for a one-hour assessment session.

Participants

A total of 144 community-dwelling older adults over 60 years old were eligible to participate in the study. Exclusion criteria were as follows: older adults with urinary infection, acute prostatic injury, genital prolapse, psychiatric disorder (i.e., Alzheimer's disease), and severe visual, auditory, or cognitive impairment that compromised their capability to complete surveys. Participants were recruited using a convenience sampling method from March to November 2018 by a physical therapist who did not take part in the study. The study included community-dwelling older adults who agreed to participate voluntarily in the study. The researchers invited potential participants, explained the study procedures, and answered questions before the older adults agreed and gave their consent to participate.

Measures

Participants were requested to supply various data, such as demographic information, anthropometric measures, and self-reported measures. We developed an assessment form to register demographic, anthropometric, and clinical data. These measures were recollected by two physical therapists. The data included age (in years), sex (female or male), weight (in kilograms), height (in centimeters), residence (urban or rural), presence of comorbidities (no or yes), medications (number), urinary status (International Consultation on Incontinence Questionnaire-Short Form (ICIQ-SF) score, points), cognitive function (Mini-Mental State Examination (MMSE), points), and activity of daily living (ADL) functionality (Barthel score, points).

Dependent variable: UI

Self-reported UI was assessed through the ICIQ-SF [14]. The initial two questions of the ICIQ-SF address how often and how many times urine leakage occurred over the past four weeks. The third question evaluates the extent to which these leaks impact daily living. The ICIQ-SF score is determined based on responses to these three questions and can range from 0 to 21 points. The ICIQ-SF includes a series of questions about the circumstances in which urine leakage occurs. We drew a distinction between stress UI (urine "leaks when you cough or sneeze" or "leaks when you are physically active/exercising"), urge UI (urine "leaks before you can get to the toilet"), and mixed UI (presence of both) [14]. In this study, we defined older adults as continent if their score was zero and incontinent if they received any score above zero [15]. UI severity was categorized as slight (1-5 points), moderate (6-12 points), severe (13-18 points), or very severe (19-21 points) based on the classification proposed by Klovning et al. (2009) [15]. The ICIQ-SF has proven to be a robust brief questionnaire, with an "acceptable" convergent validity, a "good" reliability, and a "moderate" to "very good" stability in test-retest analysis with a Cronbach's alpha of 0.95 [14].

Independent variables

The independent variables were demographic data (age, sex, and residence), anthropometric measures (weight, height, and body mass index (BMI)), and self-reported measures (comorbidities, polypharmacy, cognitive status, and ADL functionality). Polypharmacy is generally defined as the use of multiple medications by an older adult and is defined as the use of five or more medications [16]. The MMSE is a widely used cognitive screening tool for assessing cognitive impairment in older adults. It evaluates orientation, memory, attention, language, and practice and demonstrates good construct validity and specificity (80%-100%) [16]. The ADL functionality was evaluated with the Barthel score, which is a widely used measure of functional ability in the older adult population. It assesses independence in 10 activities of daily living, with scores ranging from 0 to 100 [17]. Higher scores indicate greater independence, with 60 being a pivotal point where patients transition from dependency to assisted independence [17].

Data collection

From March to November 2018, two physical therapists who had undergone rigorous training obtained demographic, anthropometric, and clinical data from every participant through in-person evaluation. To ensure consistency in assessments and compliance with evaluation protocols, all assessors completed a seven-hour training session before collecting data. At first, the theoretical aspects of outcome variables, constructs, instruments, and the psychometric properties for each measurement were covered over a period of three teaching hours. Following that, two hours of practical component were conducted to enhance proficiency in using the evaluation tools and interpreting the acquired measurements. Lastly, to assess the proficiency of the assessors, a two-hour practical test was conducted involving the measurement of the study outcomes.

Data analysis

Continuous variables are presented as median and interquartile range (x ± IQR), while categorical variables are shown as percentage frequency (%). The prevalence of UI was determined by taking the number of patients with UI, dividing it by the total number of patients in the sample, and then multiplying the result by 100 to express it as a percentage. Data normality was assessed using the Kolmogorov-Smirnov test. Evaluation of differences between participants with and without UI was conducted using the Mann-Whitney U test for variables because the distribution of the continuous variables was not normal. For categorical variables, difference analysis was performed using the Chi-square test.

A binary logistic regression was employed to evaluate the correlation and contribution of demographic, anthropometric, and clinical factors (independent variables) on the prevalence of UI in participants (dependent variable). To determine which variables should be incorporated into the regression model, the Spearman correlation coefficient (r) was utilized to measure the relationship between the prevalence of UI and the demographic (age), anthropometric (weight, height, and BMI), and self-reported variables of the participants (MMSE and Barthel scores). The Chi-square test was applied to evaluate the association between the presence of UI and binary categorical variables (i.e., sex, residency, and presence of comorbidities). Cramer's V test was employed to evaluate the association between the presence of UI and ordinal categorical variables (i.e., age, BMI, and severity categories). Variables were considered for inclusion in the models if their correlation with the prevalence of UI was statistically significant, indicated by p-values less than 0.05. The correlation strength was categorized as follows: very strong (r > 0.90), strong (r between 0.70 and 0.89), moderate (r between 0.50 and 0.69), weak (r between 0.30 and 0.49), and negligible (r < 0.30) [18]. A significance level of 5% was applied to all analyses, which were conducted utilizing IBM SPSS Statistics software version 25.0 (IBM Corp., Armonk, NY).

Ethics approval

The study protocol has been examined and approved by the Scientific Ethics Committee of Mayor University, which is officially accredited by the Chilean Ministry of Health (protocol number: 17651). All procedures were conducted in accordance with the ethical standards outlined in the Declaration of Helsinki. Informed consent was obtained from all participants prior to their inclusion in the study. The consent process involved a detailed explanation of the study's objectives, procedures, potential risks, and benefits, ensuring that participants fully understood their involvement. We took measures to minimize potential risks to participants. These included ensuring privacy during data collection, using non-invasive methods, and offering participants the option to withdraw from the study at any time without repercussions. Additionally, sensitivity and respect were maintained when discussing topics related to incontinence, recognizing the potential emotional impact on participants. Finally, once deidentified, the data will be stored in a secure database with daily backups, and physical copies will be maintained in an authorized secure storage facility.

## Results

Demographic, anthropometric, and self-reported measures

Out of 144 community-dwelling older adults who were invited, 36 were found ineligible, and 108 agreed to participate in the study. The median age of the participants was 69.0 ± 8.0 years, 84 (77.8%) were women, and 71 (65.7%) lived in an urban residence. Of the total number of older adults, 36 (33.3%) were in the 65-69 and 26 (24.1%) were in the 70-74 age ranges. Regarding anthropometric characteristics, 56 (51.9%) participants had an obese BMI category. Every participant had coexisting medical conditions. In relation to this, 77 (71.3%), 44 (40%), and 40 (37%) participants reported arterial hypertension, diabetes mellitus, and osteoarthritis, respectively. Polypharmacy was present in 36 (33.3%) older adults. Furthermore, cognitive function in the MMSE was 17.0 ± 4.0 points, and ADL functionality in the Barthel score was 100.0 ± 0.0 points. In Table 1, a summary of the demographic, anthropometric, and self-reported measures of the participants is presented.

**Table 1:** Comparison of demographic, anthropometric, and self-reported measures between older adults with and without UI UI: urinary incontinence, IQR: interquartile range, BMI: body mass index, ADL: activities of daily living, P: UI versus non-UI intergroup difference *P < 0.05, **P < 0.01 ^a^Mann-Whitney U test ^b^Chi-square test Prepared by the authors based on the results of the study

Variables	Total (N = 108)	With UI (n = 52)	Without UI (n = 56)	p-value	Mann-Whitney U test value	Chi-square test value
Demographics						
Age, years (median ± IQR)	69.0 ± 8.0	69.0 ± 9.0	69.5 ± 9.0	0.794^a^	1413.5	-
Age, range (number (%))				0.626^b^	-	3.486
60-64 years	20 (18.5)	8 (15.4)	12 (21.4)	-	-	-
65-69 years	36 (33.3)	20 (38.5)	16 (28.6)	-	-	-
70-74 years	26 (24.1)	10 (19.2)	16 (28.6)	-	-	-
75-79 years	10 (9.3)	5 (9.6)	5 (8.9)	-	-	-
80-84 years	12 (11.1)	6 (11.5)	6 (10.7)	-	-	-
85-90 years	4 (3.7)	3 (5.8)	1 (1.8)	-	-	-
Sex				0.010^*b^	-	6.623
Women (number (%))	84 (77.8)	46 (88.5)	38 (67.9)	-	-	-
Men (number (%))	24 (22.2)	6 (11.5)	18 (32.1)	-	-	-
Residency				0.196^b^	-	1.671
Urban (number (%))	71 (65.7)	31 (59.6)	40 (71.4)	-	-	-
Rural (number (%))	37 (34.3)	21 (40.4)	16 (28.6)	-	-	-
Anthropometrics						
Weight, kg (median ± IQR)	69.0 ± 16.0	69.0 ± 15.0	69.0 ± 18.0	0.696^a^	1392.5	-
Height, cm (median ± IQR)	152.0 ± 10.0	152.0 ± 10.0	153.0 ± 14.0	0.220^a^	1257.0	-
BMI (median ± IQR)	30.1 ± 5.6	30.1 ± 5.7	30.3 ± 5.9	0.588^a^	1368.0	-
Nutritional status				0.987^b^	-	0.026
Underweight (number (%))	0 (0.0)	0 (0.0)	0 (0.0)	-	-	-
Normal (number (%))	13 (12.0)	6 (11.5)	7 (12.5)	-	-	-
Overweight (number (%))	39 (36.1)	19 (36.5)	20 (35.7)	-	-	-
Obese (number (%))	56 (51.9)	27 (51.9)	29 (51.8)	-	-	-
Self-reported						
Arterial hypertension (number (%))	77 (71.3)	35 (67.3)	42 (75.0)	0.377^b^	-	0.780
Diabetes mellitus (number (%))	44 (40.7)	18 (34.6)	26 (46.4)	0.212^b^	-	1.559
Dyslipidemia (number (%))	32 (29.6)	15 (28.8)	17 (30.4)	0.864^b^	-	0.030
Chronic heart failure (number (%))	22 (20.4)	9 (17.3)	13 (23.2)	0.446^b^	-	0.580
Cancer (number (%))	2 (1.9)	1 (1.9)	1 (1.8)	0.958^b^	-	0.003
Ophthalmologic disease (number (%))	2 (1.9)	2 (3.8)	0 (0.0)	0.139^b^	-	2.194
Osteoarthritis (number (%))	40 (37.0)	15 (28.8)	25 (44.6)	0.089^b^	-	2.885
Rheumatoid arthritis (number (%))	5 (4.6)	5 (9.6)	0 (0.0)	0.017^*b^	-	5.646
Hypothyroidism (number (%))	11 (10.2)	11 (21.2)	0 (0.0)	0.000^**b^	-	13.190
Depression (number (%))	3 (2.8)	2 (3.8)	1 (1.8)	0.515^b^	-	0.424
Polypharmacy (number (%))	36 (33.3)	17 (32.7)	19 (33.9)	0.892^b^	-	0.019
Cognitive function, points (median ± IQR)	17.0 ± 4.0	18.0 ± 4.0	17.0 ± 5.0	0.780^a^	1411.5	-
ADL functionality, points (median ± IQR)	100.0 ± 0.0	100.0 ± 0.0	100.0 ± 0.0	0.423^a^	1385.0	-

UI characteristics and differences between older adults with and without UI

The prevalence of UI in the total sample was 48.1% (n = 52). Stress, urge, and mixed types of UI were present in 75 (69.4%), 15 (14.3%), and 18 (16.3%) participants, respectively. Female sex, rheumatoid arthritis, and hypothyroidism were significantly more frequent in older adults with UI (P < 0.05). No significant differences were found in anthropometric measures between older adults with and without UI (P > 0.05). The demographic, anthropometric, and self-reported measures between older adults with and without UI are shown in Table 1.

Associated factors

Correlation coefficients showed a significantly weak positive correlation between the prevalence of UI and hypothyroidism (r = 0.35; P < 0.01). Furthermore, a significantly negligible positive correlation was established between the prevalence of UI and sex (r = 0.25; P < 0.05) and rheumatoid arthritis (r = 0.23; P < 0.05). In addition, the binary logistic regression analysis that investigated the predictors of UI presence found that the female sex and the presence of rheumatoid arthritis and hypothyroidism increases the risk of UI three times (β = 3.1; 95% confidence interval (CI) = 1.05-9.34; P = 0.041).

## Discussion

The purpose of this study was to assess the prevalence and association between UI and a variety of demographic, anthropometric, and self-reported measures in Chilean community-dwelling older adults. In this context, the UI was 48.1% in this sample and predominantly in older women. Correlations ranging from negligible to weak were observed between the presence of UI and the female sex, rheumatoid arthritis, and hypothyroidism. Finally, our regression analysis showed that the female sex, rheumatoid arthritis, and hypothyroidism increased the risk of sarcopenia three times.

UI characteristics

Our results align with earlier research investigating the prevalence of UI in older adults [12,19]. Recently, a systematic review with meta-analysis by Batmani et al. (2021) showed a worldwide older adult UI prevalence of 37.1% [12]. The study by Hunskaar et al. (2004) reported a prevalence of UI of 48.7% in 17,080 older European adults [19]. Furthermore, UI is a more common condition in women. The National Health and Nutritional Examination Survey (NHANES) 2001-2008 cycles found that UI prevalence in the United States is much higher in women than in men (51.1% versus 13.9%, respectively) [2], which is consistent with the UI prevalence in women in our study (54.8%). On the other hand, stress UI is the most common type of UI in older adults, particularly women [20]. Age-related changes in the lower urinary tract, including decreased bladder capacity and reduced maximum urethral closure pressure, contribute to its prevalence [21]. In this line, our study found that stress UI was present in 69.4% of older adults. In addition, UI is attributed to the failure of the pelvic floor muscles and supporting structures to maintain continence under increased abdominal pressure. In this sense, obesity can exacerbate the risk of developing stress UI, as it contributes to increased intra-abdominal pressure and strain on the pelvic floor [22]. In our cohort, 87.5% of older adults were overweight or obese, which could have contributed to the high prevalence of stress-related UI. However, due to the multifactorial nature of UI, more research is needed to understand the interaction of these factors in older adults.

Differences between older adults with and without UI

Research indicates significant differences between older adults with and without UI with respect to osteoarthritis, depression, and cognitive function [23-27]. However, our findings do not align with these reports, possibly due to differences in nutritional status and comorbidity presence. Celik et al. (2024) demonstrated that individuals with UI have a higher prevalence of osteoarthritis compared to those without, with mobility impairment and difficulties with daily activity further increasing the risk of UI [23]. Therefore, UI significantly affects the mental health of older adults. In this sense, individuals with UI are more likely to experience depression compared to those without [24]. Additionally, the severity and frequency of UI symptoms are positively correlated with depression scores [25]. Furthermore, significant differences in cognitive function have been observed between older adults with and without UI. For example, Huang et al. (2007) reported that cognitive decline in older women was correlated with increased reports of UI that interfered with daily activities, suggesting that cognitive impairment may hinder the ability to cope with incontinence [26]. Furthermore, Hatta et al. (2011) found that UI was associated with deficits in attention and information processing speed, but not memory or visuospatial function [27]. Collectively, these studies underscore the multifaceted and bidirectional impact of UI on older adults' physical and mental well-being.

UI-associated factors

UI is prevalent in at least 25% of the adult population [3]. Our findings suggest an even higher prevalence, consistent with data from the NHANES survey in the United States [2], probably due to epidemiological similarities and the older age group studied. Age-related factors and social determinants of health significantly influence UI prevalence, as older age is strongly associated with higher rates of the condition [9]. In women, UI is driven by hormonal changes related to menopause, multiparity, and obesity, which affect the pelvic floor [7]. Additionally, many patients with UI do not report symptoms during routine medical consultations, often due to modesty or shame, which is further exacerbated in populations with high social and economic deprivation [28]. Our study was conducted on older adults with advanced demographic transition, multidimensional poverty, limited access to healthcare, and educational barriers. These factors may perpetuate and worsen UI in this population.

Strength and limitations

To our knowledge, this study would be one of the few studies in Chile that investigated the impact of UI in the older adult population. This study would serve as one of the few sources of information about the prevalence and impacts of UI in Chile.

There are several limitations in our study. First, the UI diagnosis was based on the self-reported ICIQ-SF questionnaire without laboratory studies. This could overestimate the prevalence of UI in the older adult sample. However, ICIQ-SF has shown a significant correlation with urodynamic findings [29], which could mitigate this aspect. Second, the sample size may limit the detection of additional factors associated with UI. However, the results remain valid, as they are comparable with clinically and epidemiologically established findings from other studies. Third, the cross-sectional design in our study does not allow us to establish causal inferences between the UI and the rest of the variables. In this sense, future studies with a more extensive sample and multicentric design are necessary.

## Conclusions

In a sample of community-dwelling older adults, UI was present in 48.1% of the participants. The prevalence of UI was correlated with the female sex, rheumatoid arthritis, and hypothyroidism. This study was conducted on older adults with advanced demographic transition, multidimensional poverty, limited access to healthcare, and educational barriers. These factors may perpetuate and worsen UI in this population. In addition, UI has a multifaceted impact on older adults' physical and mental well-being. More efforts are needed to address the risk factors associated with UI, which could significantly improve the quality of life of Chilean older adults living in the community.
